# *catena*-Poly[[(1,10-phenanthroline-κ^2^ *N*,*N*′)lead(II)]-μ-azido-κ^2^ *N* ^1^:*N* ^3^-μ-nitrito-κ^3^ *O*,*O*′:*O*′-[(1,10-phenanthroline-κ^2^ *N*,*N*′)lead(II)]-di-μ-azido-κ^4^ *N* ^1^:*N* ^1^]

**DOI:** 10.1107/S1600536810022907

**Published:** 2010-06-18

**Authors:** Gholamhossein Mohammadnezhad, Ali Reza Ghanbarpour, Mostafa M. Amini, Seik Weng Ng

**Affiliations:** a Department of Chemistry, General Campus, Shahid Beheshti University, Tehran 1983963113, Iran; b Department of Chemistry, University of Malaya, 50603 Kuala Lumpur, Malaysia

## Abstract

The title coordination polymer, [Pb_2_(N_3_)_3_(NO_2_)(C_12_H_8_N_2_)_2_]_*n*_, has as the repeat unit a centrosymmetric dinuclear mol­ecule having azide and nitrite groups that bridge adjacent heterocycle-coordinated metal centers. One of the azide group uses its terminal ends to bridge whereas the nitrite group chelates to one metal atom and uses one of its O atoms to bridge. The azide and nitrite groups are disordered with respect to each other in a 1:1 ratio. Adjacent dinuclear mol­ecules are further bridged by the other two azide groups, generating a linear chain motif parallel to [010]. Half of the Pb atoms show a Ψ-dodeca­hedral coordination and the other half show a Ψ-penta­gonal-bipyramidal coordination.

## Related literature

For the crystal structure of a related lead azide complex, see: Marandi *et al.* (2007 ▶).
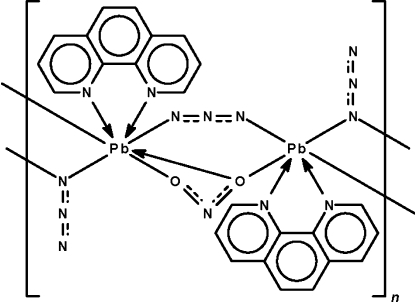


## Experimental

### 

#### Crystal data

[Pb_2_(N_3_)_3_(NO_2_)(C_12_H_8_N_2_)_2_]
*M* *_r_* = 946.89
Triclinic, 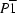

*a* = 7.6860 (5) Å
*b* = 9.2056 (6) Å
*c* = 9.9080 (7) Å
α = 90.541 (1)°
β = 109.665 (1)°
γ = 104.626 (1)°
*V* = 635.32 (7) Å^3^
*Z* = 1
Mo *K*α radiation
μ = 13.29 mm^−1^
*T* = 100 K
0.30 × 0.30 × 0.30 mm

#### Data collection

Bruker SMART APEX diffractometer
Absorption correction: multi-scan (*SADABS*; Sheldrick, 1996 ▶) *T*_min_ = 0.040, *T*_max_ = 0.109
6004 measured reflections
2888 independent reflections
2716 reflections with *I* > 2σ(*I*)
*R*_int_ = 0.033

#### Refinement

*R*[*F* ^2^ > 2σ(*F* ^2^)] = 0.028
*wR*(*F* ^2^) = 0.071
*S* = 1.03
2888 reflections
193 parameters
12 restraints
H-atom parameters constrained
Δρ_max_ = 2.17 e Å^−3^
Δρ_min_ = −1.97 e Å^−3^

### 

Data collection: *APEX2* (Bruker, 2009 ▶); cell refinement: *SAINT* (Bruker, 2009 ▶); data reduction: *SAINT*; program(s) used to solve structure: *SHELXS97* (Sheldrick, 2008 ▶); program(s) used to refine structure: *SHELXL97* (Sheldrick, 2008 ▶); molecular graphics: *X-SEED* (Barbour, 2001 ▶); software used to prepare material for publication: *publCIF* (Westrip, 2010 ▶).

## Supplementary Material

Crystal structure: contains datablocks global, I. DOI: 10.1107/S1600536810022907/xu2775sup1.cif

Structure factors: contains datablocks I. DOI: 10.1107/S1600536810022907/xu2775Isup2.hkl

Additional supplementary materials:  crystallographic information; 3D view; checkCIF report

## Figures and Tables

**Fig. 1.**
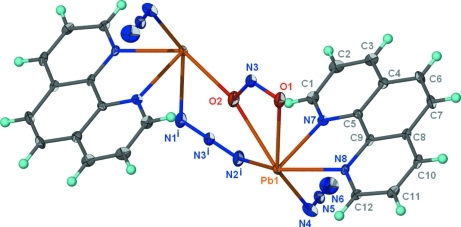
Thermal ellipsoid plot (Barbour, 2001) of Pb2(N3)3(NO2)(C12H8NO)2 at the 70% probability level; hydrogen atoms are drawn as spheres of arbitrary radius. Symmetry code: i = 1 - x, 1 - y, 1 - z.

## supplementary crystallographic information

### Comment

There are a number of 1,10-phenanthroline-chelated lead(II) compounds having inorganic anions (having only few atoms) as counterions whose crystal structures have been reported. For some, two counterions exist in the crystal structure that originally came from the reactants used in the synthesis.

The azide derivative is a polymeric dinuclear chain compound in which the lead atoms show PbN_8_ and PbN_6_O_2_ dodecahedral coordination. In lead azide nicotinate, the azide unit engages in µ_3_ bridging (Marandi *et al.*, 2007). In Pb_2_(N_3_)_3_(NO_2_)(C_12_H_8_NO)_2 _(Scheme I, Fig. 1), the azide groups bridge adjacent heterocycle-coordinated metal centers through one nitrogen atom and the third bridging through two nitrogen atoms. The nitrite group chelates to one metal atom and uses one oxygen atom to bind to the inversion-related lead atom. The bridging interactions lead to the formation of a linear chain motif. One of the azide groups that uses its terminal nitrogen atoms to bridge is disordered with respect to the nitrite group in a 1:1 ratio. The disorder gives rise to a *Ψ*-dodecahedral geometry for 50% of the lead atoms and a *Ψ*-pentagonal bipyramidal geometry for the other 50% of the lead atoms.

### Experimental

1,10-Phenanthroline (0.36 g, 2 mmol) and sodium azide (0.13 g, 1 mmol) were placed in one arm of a convection tube, and lead(II) nitrate (0.33 g, 1 mmol) and sodium nitrite (0.07 g, 1 mmol) in the other. Methanol was then added to fill both arms and the tube was sealed. The ligand-containing arm was immersed in an oil bath at 333 K, whereas the other was left at ambient temperature. After 1 day, crystals deposited in the arm that was kept at ambient temperature.

### Refinement

Carbon-bound H-atoms were placed in calculated positions [C–H = 0.95 Å; *U*(H) = 1.2*U*_eq_(C)] in the riding model approximation.

One of the two azide ions (N1, N2, N3) is disordered with respect to a nitrite ion (O1, O2, N3); the N3 atom is ordered. The N1 and O1 atoms occupy the same site; the atoms are give half occupancy and the same temperature factors. The N2 atom is disordered with respect to the O2 atom but they do not occupy the same site; their temperature factors were also restrained to be identical.

The final difference fourier map had a large peak/deep hole in the vicinity of the lead atom.

### Crystal data

**Table:**

[Pb_2_(N_3_)_3_(NO_2_)(C_12_H_8_N_2_)_2_]	*Z* = 1
*M**_r_* = 946.89	*F*(000) = 438
Triclinic, *P*1	*D*_x_ = 2.475 Mg m^−^^3^
Hall symbol: -P 1	Mo *K*α radiation, λ = 0.71073 Å
*a* = 7.6860 (5) Å	Cell parameters from 4831 reflections
*b* = 9.2056 (6) Å	θ = 2.3–28.3°
*c* = 9.9080 (7) Å	µ = 13.29 mm^−^^1^
α = 90.541 (1)°	*T* = 100 K
β = 109.665 (1)°	Irregular, yellow
γ = 104.626 (1)°	0.30 × 0.30 × 0.30 mm
*V* = 635.32 (7) Å^3^	

### Data collection

**Table:**

Bruker SMART APEX diffractometer	2888 independent reflections
Radiation source: fine-focus sealed tube	2716 reflections with *I* > 2σ(*I*)
graphite	*R*_int_ = 0.033
ω scans	θ_max_ = 27.5°, θ_min_ = 2.2°
Absorption correction: multi-scan (*SADABS*; Sheldrick, 1996)	*h* = −9→9
*T*_min_ = 0.040, *T*_max_ = 0.109	*k* = −11→11
6004 measured reflections	*l* = −12→12

### Refinement

**Table:**

Refinement on *F*^2^	Primary atom site location: structure-invariant direct methods
Least-squares matrix: full	Secondary atom site location: difference Fourier map
*R*[*F*^2^ > 2σ(*F*^2^)] = 0.028	Hydrogen site location: inferred from neighbouring sites
*wR*(*F*^2^) = 0.071	H-atom parameters constrained
*S* = 1.03	*w* = 1/[σ^2^(*F*_o_^2^) + (0.0348*P*)^2^ + 1.3333*P*] where *P* = (*F*_o_^2^ + 2*F*_c_^2^)/3
2888 reflections	(Δ/σ)_max_ = 0.001
193 parameters	Δρ_max_ = 2.17 e Å^−^^3^
12 restraints	Δρ_min_ = −1.97 e Å^−^^3^

### Fractional atomic coordinates and isotropic or equivalent isotropic displacement parameters (Å^2^)

**Table:**

	*x*	*y*	*z*	*U*_iso_*/*U*_eq_	Occ. (<1)
Pb1	0.56829 (3)	0.79283 (2)	0.561361 (19)	0.00881 (8)	
O1	0.7795 (7)	0.6258 (5)	0.7489 (5)	0.0169 (9)	0.50
O2	0.5962 (13)	0.4948 (9)	0.5579 (9)	0.0197 (14)	0.50
N1	0.7795 (7)	0.6258 (5)	0.7489 (5)	0.0169 (9)	0.50
N2	0.7445 (16)	0.3876 (12)	0.6296 (11)	0.0197 (14)	0.50
N3	0.7470 (8)	0.5047 (5)	0.6855 (5)	0.0156 (10)	
N4	0.3085 (7)	0.9214 (6)	0.5147 (5)	0.0168 (10)	
N5	0.1522 (7)	0.8609 (5)	0.5166 (5)	0.0136 (10)	
N6	−0.0006 (8)	0.8055 (6)	0.5194 (6)	0.0260 (12)	
N7	0.4053 (6)	0.6818 (5)	0.7324 (5)	0.0090 (9)	
N8	0.6931 (7)	0.9476 (5)	0.8019 (5)	0.0085 (9)	
C1	0.2609 (8)	0.5559 (6)	0.6968 (6)	0.0124 (11)	
H1	0.2033	0.5142	0.5988	0.015*	
C2	0.1915 (8)	0.4829 (6)	0.7996 (7)	0.0148 (11)	
H2	0.0900	0.3924	0.7718	0.018*	
C3	0.2717 (8)	0.5438 (6)	0.9397 (6)	0.0138 (11)	
H3	0.2254	0.4958	1.0101	0.017*	
C4	0.4224 (8)	0.6771 (6)	0.9804 (6)	0.0106 (10)	
C5	0.4849 (8)	0.7444 (6)	0.8717 (6)	0.0084 (10)	
C6	0.5120 (8)	0.7478 (6)	1.1264 (6)	0.0131 (11)	
H6	0.4726	0.7008	1.2002	0.016*	
C7	0.6505 (8)	0.8794 (7)	1.1603 (6)	0.0132 (11)	
H7	0.7065	0.9244	1.2572	0.016*	
C8	0.7146 (7)	0.9524 (6)	1.0510 (6)	0.0080 (10)	
C9	0.6343 (7)	0.8846 (6)	0.9074 (6)	0.0081 (10)	
C10	0.8545 (8)	1.0937 (6)	1.0810 (6)	0.0110 (11)	
H10	0.9108	1.1433	1.1762	0.013*	
C11	0.9080 (8)	1.1579 (6)	0.9729 (6)	0.0115 (11)	
H11	1.0002	1.2532	0.9911	0.014*	
C12	0.8231 (8)	1.0797 (6)	0.8329 (6)	0.0093 (10)	
H12	0.8615	1.1243	0.7577	0.011*	

### Atomic displacement parameters (Å^2^)

**Table:**

	*U*^11^	*U*^22^	*U*^33^	*U*^12^	*U*^13^	*U*^23^
Pb1	0.01002 (12)	0.00884 (11)	0.00691 (11)	0.00024 (7)	0.00383 (8)	−0.00010 (7)
O1	0.017 (2)	0.016 (2)	0.017 (2)	0.0021 (18)	0.0072 (19)	−0.0037 (17)
O2	0.024 (4)	0.018 (3)	0.017 (3)	0.004 (3)	0.009 (3)	−0.004 (2)
N1	0.017 (2)	0.016 (2)	0.017 (2)	0.0021 (18)	0.0072 (19)	−0.0037 (17)
N2	0.024 (4)	0.018 (3)	0.017 (3)	0.004 (3)	0.009 (3)	−0.004 (2)
N3	0.021 (3)	0.014 (2)	0.012 (2)	0.005 (2)	0.006 (2)	−0.0002 (18)
N4	0.011 (3)	0.025 (3)	0.017 (3)	0.007 (2)	0.006 (2)	0.008 (2)
N5	0.015 (3)	0.017 (2)	0.010 (2)	0.006 (2)	0.0042 (19)	0.0014 (18)
N6	0.017 (3)	0.027 (3)	0.031 (3)	0.002 (2)	0.007 (2)	0.004 (2)
N7	0.006 (2)	0.010 (2)	0.011 (2)	0.0017 (17)	0.0026 (18)	0.0018 (17)
N8	0.009 (2)	0.008 (2)	0.007 (2)	0.0025 (17)	0.0007 (18)	−0.0018 (16)
C1	0.008 (3)	0.012 (3)	0.014 (3)	−0.001 (2)	0.003 (2)	−0.002 (2)
C2	0.007 (3)	0.012 (3)	0.023 (3)	−0.002 (2)	0.006 (2)	0.002 (2)
C3	0.010 (3)	0.013 (3)	0.022 (3)	0.003 (2)	0.010 (2)	0.004 (2)
C4	0.010 (3)	0.012 (3)	0.013 (3)	0.006 (2)	0.005 (2)	0.002 (2)
C5	0.008 (3)	0.010 (2)	0.007 (2)	0.004 (2)	0.003 (2)	0.0022 (19)
C6	0.016 (3)	0.017 (3)	0.010 (3)	0.004 (2)	0.008 (2)	0.003 (2)
C7	0.014 (3)	0.020 (3)	0.008 (3)	0.009 (2)	0.004 (2)	0.001 (2)
C8	0.003 (2)	0.011 (2)	0.010 (3)	0.0033 (19)	0.002 (2)	−0.0006 (19)
C9	0.005 (3)	0.010 (2)	0.010 (3)	0.0045 (19)	0.002 (2)	−0.0016 (19)
C10	0.007 (3)	0.014 (3)	0.011 (3)	0.005 (2)	0.001 (2)	−0.004 (2)
C11	0.007 (3)	0.008 (2)	0.016 (3)	0.0001 (19)	0.002 (2)	−0.002 (2)
C12	0.006 (3)	0.009 (2)	0.013 (3)	0.0025 (19)	0.004 (2)	0.0006 (19)

### Geometric parameters (Å, °)

**Table:**

Pb1—N4	2.487 (5)	C1—H1	0.9500
Pb1—N7	2.510 (4)	C2—C3	1.363 (8)
Pb1—N8	2.517 (4)	C2—H2	0.9500
Pb1—N2^i^	2.642 (11)	C3—C4	1.403 (8)
Pb1—O2^i^	2.693 (8)	C3—H3	0.9500
Pb1—N4^ii^	2.764 (5)	C4—C5	1.410 (8)
O1—N3	1.200 (6)	C4—C6	1.441 (8)
O2—N3	1.382 (10)	C5—C9	1.442 (7)
O2—Pb1^i^	2.693 (8)	C6—C7	1.347 (8)
N2—N3	1.200 (11)	C6—H6	0.9500
N2—Pb1^i^	2.642 (11)	C7—C8	1.440 (8)
N4—N5	1.196 (7)	C7—H7	0.9500
N4—Pb1^ii^	2.764 (5)	C8—C9	1.412 (7)
N5—N6	1.166 (7)	C8—C10	1.417 (7)
N7—C1	1.334 (7)	C10—C11	1.360 (8)
N7—C5	1.360 (7)	C10—H10	0.9500
N8—C12	1.321 (7)	C11—C12	1.416 (7)
N8—C9	1.353 (7)	C11—H11	0.9500
C1—C2	1.404 (8)	C12—H12	0.9500
			
N4—Pb1—N7	78.10 (15)	C3—C2—C1	119.1 (5)
N4—Pb1—N8	82.36 (16)	C3—C2—H2	120.4
N7—Pb1—N8	66.32 (15)	C1—C2—H2	120.4
N4—Pb1—N2^i^	72.7 (3)	C2—C3—C4	120.3 (5)
N7—Pb1—N2^i^	81.9 (3)	C2—C3—H3	119.9
N8—Pb1—N2^i^	143.0 (2)	C4—C3—H3	119.9
N4—Pb1—O2^i^	107.5 (2)	C3—C4—C5	117.6 (5)
N7—Pb1—O2^i^	78.2 (2)	C3—C4—C6	123.0 (5)
N8—Pb1—O2^i^	140.5 (2)	C5—C4—C6	119.4 (5)
N2^i^—Pb1—O2^i^	36.6 (3)	N7—C5—C4	121.6 (5)
N4—Pb1—N4^ii^	70.67 (18)	N7—C5—C9	118.7 (5)
N7—Pb1—N4^ii^	135.88 (14)	C4—C5—C9	119.6 (5)
N8—Pb1—N4^ii^	79.09 (15)	C7—C6—C4	121.3 (5)
N2^i^—Pb1—N4^ii^	116.0 (3)	C7—C6—H6	119.4
O2^i^—Pb1—N4^ii^	140.4 (2)	C4—C6—H6	119.4
N3—O2—Pb1^i^	111.5 (5)	C6—C7—C8	120.5 (5)
N3—N2—Pb1^i^	123.2 (7)	C6—C7—H7	119.7
O1—N3—N2	170.0 (8)	C8—C7—H7	119.7
O1—N3—O2	107.1 (5)	C9—C8—C10	117.7 (5)
N2—N3—O2	80.7 (7)	C9—C8—C7	119.9 (5)
N5—N4—Pb1	123.2 (4)	C10—C8—C7	122.4 (5)
N5—N4—Pb1^ii^	127.3 (4)	N8—C9—C8	121.9 (5)
Pb1—N4—Pb1^ii^	109.33 (18)	N8—C9—C5	118.9 (5)
N6—N5—N4	178.2 (6)	C8—C9—C5	119.2 (5)
C1—N7—C5	119.3 (5)	C11—C10—C8	119.7 (5)
C1—N7—Pb1	123.0 (4)	C11—C10—H10	120.2
C5—N7—Pb1	117.2 (3)	C8—C10—H10	120.2
C12—N8—C9	119.0 (4)	C10—C11—C12	118.6 (5)
C12—N8—Pb1	123.5 (3)	C10—C11—H11	120.7
C9—N8—Pb1	117.2 (3)	C12—C11—H11	120.7
N7—C1—C2	122.0 (5)	N8—C12—C11	123.0 (5)
N7—C1—H1	119.0	N8—C12—H12	118.5
C2—C1—H1	119.0	C11—C12—H12	118.5
			
Pb1^i^—N2—N3—O1	−169 (4)	N7—C1—C2—C3	1.1 (9)
Pb1^i^—N2—N3—O2	−27.1 (7)	C1—C2—C3—C4	−0.4 (8)
Pb1^i^—O2—N3—O1	−162.7 (4)	C2—C3—C4—C5	0.4 (8)
Pb1^i^—O2—N3—N2	23.7 (7)	C2—C3—C4—C6	179.4 (5)
N7—Pb1—N4—N5	−36.2 (4)	C1—N7—C5—C4	1.9 (8)
N8—Pb1—N4—N5	−103.5 (5)	Pb1—N7—C5—C4	−170.4 (4)
N2^i^—Pb1—N4—N5	48.8 (5)	C1—N7—C5—C9	−176.9 (5)
O2^i^—Pb1—N4—N5	37.2 (5)	Pb1—N7—C5—C9	10.8 (6)
N4^ii^—Pb1—N4—N5	175.4 (6)	C3—C4—C5—N7	−1.2 (8)
N7—Pb1—N4—Pb1^ii^	148.4 (2)	C6—C4—C5—N7	179.8 (5)
N8—Pb1—N4—Pb1^ii^	81.06 (19)	C3—C4—C5—C9	177.6 (5)
N2^i^—Pb1—N4—Pb1^ii^	−126.5 (3)	C6—C4—C5—C9	−1.4 (8)
O2^i^—Pb1—N4—Pb1^ii^	−138.2 (2)	C3—C4—C6—C7	−177.0 (6)
N4^ii^—Pb1—N4—Pb1^ii^	0.0	C5—C4—C6—C7	2.0 (8)
N4—Pb1—N7—C1	90.6 (4)	C4—C6—C7—C8	−0.7 (8)
N8—Pb1—N7—C1	177.4 (5)	C6—C7—C8—C9	−1.2 (8)
N2^i^—Pb1—N7—C1	16.7 (5)	C6—C7—C8—C10	177.5 (5)
O2^i^—Pb1—N7—C1	−20.3 (4)	C12—N8—C9—C8	−3.1 (7)
N4^ii^—Pb1—N7—C1	135.9 (4)	Pb1—N8—C9—C8	171.4 (4)
N4—Pb1—N7—C5	−97.4 (4)	C12—N8—C9—C5	176.7 (5)
N8—Pb1—N7—C5	−10.6 (3)	Pb1—N8—C9—C5	−8.8 (6)
N2^i^—Pb1—N7—C5	−171.3 (4)	C10—C8—C9—N8	2.7 (7)
O2^i^—Pb1—N7—C5	151.7 (4)	C7—C8—C9—N8	−178.6 (5)
N4^ii^—Pb1—N7—C5	−52.1 (4)	C10—C8—C9—C5	−177.0 (5)
N4—Pb1—N8—C12	−95.5 (4)	C7—C8—C9—C5	1.7 (7)
N7—Pb1—N8—C12	−175.8 (4)	N7—C5—C9—N8	−1.3 (7)
N2^i^—Pb1—N8—C12	−142.9 (5)	C4—C5—C9—N8	179.9 (5)
O2^i^—Pb1—N8—C12	156.2 (4)	N7—C5—C9—C8	178.4 (5)
N4^ii^—Pb1—N8—C12	−23.8 (4)	C4—C5—C9—C8	−0.4 (7)
N4—Pb1—N8—C9	90.3 (4)	C9—C8—C10—C11	−0.6 (7)
N7—Pb1—N8—C9	10.0 (3)	C7—C8—C10—C11	−179.3 (5)
N2^i^—Pb1—N8—C9	42.9 (6)	C8—C10—C11—C12	−0.9 (8)
O2^i^—Pb1—N8—C9	−18.0 (5)	C9—N8—C12—C11	1.4 (8)
N4^ii^—Pb1—N8—C9	161.9 (4)	Pb1—N8—C12—C11	−172.7 (4)
C5—N7—C1—C2	−1.8 (8)	C10—C11—C12—N8	0.6 (8)
Pb1—N7—C1—C2	170.0 (4)		

Symmetry codes: (i) −*x*+1, −*y*+1, −*z*+1; (ii) −*x*+1, −*y*+2, −*z*+1.
